# Is the diagnostic validity of conventional radiography for Lisfranc injury acceptable?

**DOI:** 10.1186/s13047-023-00608-0

**Published:** 2023-03-01

**Authors:** Cheng Chen, JianTao Jiang, Cheng Wang, Jian Zou, ZhongMin Shi, YunFeng Yang

**Affiliations:** 1 Department of Orthopedics, Tongji Hospital, School of Medicine, Tongji University, Shanghai, 200092 China; 2 Department of Orthopaedics, Shanghai Jiao Tong University Affiliated Sixth Peoples Hospital, Shanghai, 200233 China; 3 Department of Orthopedics, Shaoxing Shangyu Hospital of Chinese Medicine, Shaoxing, 312000 China

**Keywords:** Lisfranc injury, Diagnosis, Conventional radiographs, Validity, Clinical decision-making

## Abstract

**Background:**

Lisfranc injuries mainly involve the tarsometatarsal joint complex and are commonly misdiagnosed or missed in clinical settings. Most medical institutions prefer to use conventional radiography. However, existing studies on conventional radiographs in Lisfranc injury lack a large population-based sample, influencing the validity of the results. We aimed to determine the diagnostic validity and reliability of conventional radiography for Lisfranc injury and whether computed tomography can alter clinical decision-making.

**Methods:**

This retrospective study included 307 patients with, and 100 patients without, Lisfranc injury from January 2017 to December 2019. Diagnosis was confirmed using computed tomography. A senior and junior surgeon independently completed two assessments of the same set of anonymised conventional radiographs at least 3 months apart. The surgeons were then asked to suggest one of two treatment options (surgery or conservative treatment) for each case based on the radiographs and subsequently on the CT images.

**Results:**

All inter- and intra-observer reliabilities were moderate to very good (all κ coefficients > 0.4). The mean (range) true positive rate was 81.8% (73.9%–87.0%), true negative rate was 90.0% (85.0%–94.0%), false positive rate was 10.0% (6.0%–15.0%), false negative rate was 18.2% (13.0%–26.1%), positive predictive value was 96.1% (93.8%–97.8%), negative predictive value was 62.4% (51.5%–69.7%), classification accuracy was 83.8% (76.7%–88.2%), and balanced error rate was 14.1% (10.2%–20.5%). Three-column injuries were most likely to be recognized (mean rate, 92.1%), followed by intermediate-lateral-column injuries (mean rate, 81.5%). Medial-column injuries were relatively difficult to identify (mean rate, 60.7%). The diagnostic rate for non-displaced injuries (mean rate, 76.7%) was lower than that for displaced injuries (mean rate, 95.5%). The typical examples are given. A significant difference between the two surgeons was found in the recognition rate of non-displaced injuries (*p* = 0.005). The mean alteration rate was 21.9%; the senior surgeon tended to a lower rate (15.6%) than the junior one (28.3%) (*p* < 0.001).

**Conclusions:**

The sensitivity, specificity, and classification accuracy of conventional radiographs for Lisfranc injury were 81.8%, 90.0%, and 83.8%, respectively. Three-column or displaced injuries were most likely to be recognized. The possibility of changing the initial treatment decision after subsequently evaluating computed tomography images was 21.9%. The diagnostic and clinical decision-making of surgeons with different experience levels demonstrated some degree of variability. Protected weight-bearing and a further CT scan should be considered if a Lisfranc injury is suspected and conventional radiography is negative.

## Background

Lisfranc injuries mainly involve the tarsometatarsal joint complex [1] and are currently a trending topic in the field of foot trauma [2]. The incidence of Lisfranc injuries is reported to range from 1/60,000 to 14/100,000 person-years [3–5]. Notably, athletes may have a period of high prevalence of Lisfranc injury of up to 3/1,000 person-years [6]. Difficulties persist in the diagnosis and treatment of this injury type [7, 8], with clinical misdiagnosis and missed diagnoses often occurring. Patients with Lisfranc injuries receiving inappropriate treatment leads to chronic pain, high morbidity, and substantial disability [9, 10].

Currently, imaging examinations are the primary means of diagnosis of Lisfranc injuries [11]. Limited by cost and emergency room conditions, weight-bearing and manual stress radiography, computed tomography (CT), magnetic resonance imaging, and ultrasonography are sometimes unavailable and unfeasible. Therefore, conventional radiography remains the most commonly used imaging method as it is accessible, convenient, and cost-effective. A significant number of Lisfranc injuries, especially those with subtle initial presentations, tend to be overlooked or missed with conventional radiography [12–15]. Existing studies on the use of conventional radiography for Lisfranc injuries lack a large population sample, which may have influenced the validity of their results.

In this study, we aimed to determine the diagnostic validity and reliability of conventional radiography in Lisfranc injury diagnosis using a large sample of consecutive patients. We hypothesized that the considered treatment options of either surgery or conservative treatment may have differed based on the radiographical and subsequent CT images.

## Methods

### Patients and study design

Patients diagnosed with Lisfranc injury (*n* = 307) and non-Lisfranc injury (*n* = 100) between January 2017 and December 2019 were enrolled in this retrospective study. Patients were eligible if they were 18 to 80 years old and presented to the emergency department for an acute foot injury. Patients were excluded if they had a history of malignancy, generalized ligament laxity, paraesthesia, metabolic bone disease, or any concomitant conditions that interfered with clinical judgment.

Lisfranc injury was defined as intra-articular fractures, avulsion fractures, or joint dislocation around the tarsometatarsal joint complex. Displacement injury was defined as a bone fragment displacement or joint dislocation of > 2 mm.

All 307 diagnoses of Lisfranc injuries were confirmed by a CT scan. Among the 307 injuries, 84 (27.4%) were displaced and 223 (72.6%) were non-displaced. The remaining 100 patients were diagnosed with non-Lisfranc injury by physical examination (i.e. no pain on palpation or manipulation of the tarsometatarsal joints, no ecchymosis at the level of the midfoot) or CT scan.

The patients’ data were collected from an electronic database of medical records. CT images were independently evaluated by an experienced radiologist and foot and ankle specialist. In cases where the diagnoses differed, the case was discussed between the two specialists and a final diagnosis agreed on. Conventional radiographical images comprised non-weight-bearing foot radiographs using anteroposterior, 30° oblique, and lateral views.

Anonymised conventional radiographs for each patient were assessed by two independent foot and ankle surgeons, observer A with 6 and B with 15 years of experience. Each surgeon completed two assessments at least three months apart, and were blinded to the diagnosis. Random ranking of the imaging data was performed for each observer’s evaluation. During the second assessment, each surgeon was asked to suggest treatment options (either surgery or conservative treatment) based on the conventional radiographs. After the second assessment, the corresponding CT images were provided to each surgeon, and they were again asked to suggest treatment strategies. Whether to change the initial treatment option after evaluating CT images compared with the second conventional radiographs evaluation was recorded as qualitative variable.

This study was conducted in compliance with the principles of the Declaration of Helsinki. All patients provided informed verbal consent rather than written consent because the analysis did not require any clinical intervention and participation in the study was clearly below the minimum risk. The study protocol was approved by the ethics committee of our institution.

### Statistical analysis

Continuous variables are described as mean ± standard deviation; qualitative variables are described as numbers and proportions. Statistical analyses were performed using Microsoft Excel (version 16.15; Microsoft Corp., Redmond, WA, USA) and SPSS software (version 26.0; IBM Corp., Armonk, NY, USA). The independent sample t-test and Fisher’s exact test were used to compare the Lisfranc and non-Lisfranc injury groups. Statistical significance was set at *p* < 0.05.

Cohen’s kappa (κ), a method of evaluating reproducibility, was used to evaluate the inter- and intra-observer reliabilities. The κ coefficient was assessed according to the Landis and Koch criteria [16]: poor (0.0–0.2), fair (0.2–0.4), moderate (0.4–0.6), substantial (0.6–0.8), and almost perfect (0.8–1.0).

‘True-positive’ was defined as both ‘radiograph-positive’ and ‘CT-positive’. ‘True-negative’ was defined as both ‘radiograph-negative’ and ‘CT-negative’. ‘False-positive’ was defined as ‘radiograph-positive’ and ‘CT-negative’. ‘False-negative’ was defined as ‘radiograph-negative’ and ‘CT-positive’. Sensitivity (true positive rate) was calculated by dividing the total number of true-positive cases by that of CT-positive cases, and specificity (true negative rate) by dividing the true-negative cases by the CT-negative cases. The false positive rate was determined to be the false-positive cases divided by the CT-negative cases, and the false negative rate to be the false-negative cases divided by the CT-positive cases. The positive predictive value was calculated by dividing the true-positive cases by the radiograph-positive cases, and the negative predictive value by dividing the true-negative cases by the radiograph-negative cases [17]. The classification accuracy was obtained by dividing the true cases by all cases; the balanced error rate was obtained by taking the mean of the false positive and false negative rates.

## Results

There were 307 patients with Lisfranc injuries and 100 patients without Lisfranc injuries. We found no significant differences between the groups in terms of demographic characteristics (Table 1). Table 2 presents the results of the reliability analysis. All inter- and intra-observer reliabilities were moderate to very good (κ coefficient > 0.4). Overall, observer B had a higher κ coefficient for intra-observer reliability than observer A.

**Table 1:** Characteristics of the patients

	**Lisfranc injury** **(** ***n*** ** = 307)**	**No Lisfranc injury** **(** ***n*** ** = 100)**	***P*** **value**
Right foot, n (%)	163 (53.1%)	47 (47%)	0.290
Males, n (%)	192 (62.5%)	55 (55%)	0.180
Age, mean ± SD	46.2 ± 14.9	43.5 ± 16.4	0.118
Trauma mechanism, n (%)			0.077
Falling or slipping	98 (31.9%)	27 (27%)	-
Traffic collisions	85 (27.7%)	40 (40%)	-
Direct injury	28 (9.1%)	11 (11%)	-
Other	96 (31.3%)	22 (22%)	-

All *P* > 0.05

**Table 2:** Interobserver and intraobserver reliability

		Observer A after three months		Observer B at the first time	
		**All (** ***n*** **=407)**	**Lisfranc (** ***n=*** **307)**	**All (** ***n=*** **407)**	**Lisfranc (** ***n=*** **307)**
Observer A at the first time	**All (** ***n=*** **407)**	0.64 (0.56-0.72)	-	0.63 (0.55-0.70)	-
	**Lisfranc (** ***n=*** **307)**	-	0.51 (0.40-0.62)	-	0.41 (0.29-0.53)
Observer B after three months	**All (** ***n=*** **407)**	0.75 (0.68-0.82)	-	0.81 (0.75-0.87)	-
	**Lisfranc (** ***n=*** **307)**	-	0.58 (0.46-0.70)	-	0.61 (0.48-0.74)

The variables were described using Cohen's kappa (κ) (95% confidence interval)

All *P* < 0.01

The mean (range) true positive rate was 81.8% (73.9%–87.0%), true negative rate was 90.0% (85.0%–94.0%), false positive rate was 10.0% (6.0%–15.0%), false negative rate was 18.2% (13.0%–26.1%), positive predictive value was 96.1% (93.8%–97.8%), negative predictive value was 62.4% (51.5%–69.7%), classification accuracy was 83.8% (76.7%–88.2%), and balanced error rate was 14.1% (10.2%–20.5%) (Table 3). When the mean value of the two observations was compared between the two observers, the agreement between two observers was moderate in conventional radiographs of Lisfranc injuries (κ = 0.419, *P* < 0.001), and substantial in all conventional radiographs (κ = 0.601, *P* < 0.001).

**Table 3:** Overall results of the two observers’ two evaluations

	**Observer A**		**Observer B**		**Mean value**
	**First time**	**Three months later**	**First time**	**Three months later**	
**TPR**	227/307 (73.9%)	248/307 (80.8%)	263/307 (85.7%)	267/307 (87.0%)	81.8%
**TNR**	85/100 (85.0%)	89/100 (89.0%)	94/100 (94.0%)	92/100 (92.0%)	90.0%
**FPR**	15/100 (15.0%)	11/100 (11.0%)	6/100 (6.0%)	8/100 (8.0%)	10.0%
**FNR**	80/307 (26.1%)	59/307 (19.2%)	44/307 (14.3%)	40/307 (13.0%)	18.2%
**PPV**	227/242 (93.8%)	248/259 (95.8%)	263/269 (97.8%)	267/275 (97.1%)	96.1%
**NPV**	85/165 (51.5%)	89/148 (60.1%)	94/138 (68.1%)	92/132 (69.7%)	62.4%
**ACC**	312/407 (76.7%)	337/407 (82.8%)	357/407 (87.7%)	359/407 (88.2%)	83.8%
**BER**	20.5%	15.1%	10.2%	10.5%	14.1%
**Agreement**^**a**^	Lisfranc (*n* = 307)		κ = 0.419, *P* < 0.001		
	All (*n* = 407)		κ = 0.601, *P* < 0.001		

*TPR* True positive rate, *TNR* True negative rate, *FPR* False positive rate, *FNR* False negative rate, *PPV* Positive predictive value, *NPV* Negative predictive value, *ACC* ACC classification accuracy, *BER* Balanced error rate

^a^The mean value of Observer A’s two observations was compared to that of Observer B

There was a significant difference in the recognition rate between the two observers (*p* = 0.004) (Table 4). According to Chiodo-Myerson’s classification [18], three-column injuries had the highest likelihood of being recognized (mean rate = 92.1%), followed by intermediate-lateral-column injuries (mean rate = 81.5%). Medial-column injuries were relatively difficult to identify (mean rate = 60.7%). Although a significant difference was observed between the two observers in the recognition rate of two-column injuries (*p* = 0.037), further analysis revealed no significant difference in the subgroups. According to the displacement classification, the diagnostic rate for non-displaced injuries (mean rate = 76.7%) was lower than that for displaced injuries (mean rate = 95.5%). A significant difference was found between the two observers in the recognition rate of non-displaced injuries (*p* = 0.005).

**Table 4:** The recognition rate of Lisfranc injury in conventional radiographs

	**Observer A**		**Observer B**		**Mean value**	^*^P **value**
	**First time**	**Three months later**	**First time**	**Three months later**		
**All (** ***n*** ** = 307)**	227/307 (73.9%)	248/307 (80.8%)	263/307 (85.7%)	267/307 (87.0%)	81.8%	**0.004**^**#**^
**Chiodo-Myerson’s three-column classification** [18]						
**One column** **(** ***n*** **= 44)**	27/44 (61.4%)	32/44 (72.7%)	36/44 (81.8%)	34/44 (77.3%)	73.3%	0.171
**A (** ***n*** ** = 7)**	3/7 (42.9%)	4/7 (57.1%)	5/7 (71.4%)	5/7 (71.4%)	60.7%	0.608
**B (** ***n*** ** = 37)**	24/37 (64.9%)	28/37 (75.7%)	31/37 (83.8%)	29/37 (78.4%)	75.7%	0.278
**Two columns** **(** ***n*** **= 136)**	90/136 (66.2%)	99/136 (72.8%)	106/136 (77.9%)	113/136 (83.1%)	75.0%	**0.037**^**#**^
**AB (** ***n*** ** = 69)**	39/69 (56.5%)	48/69 (69.6%)	51/69 (73.9%)	55/69 (79.7%)	69.9%	0.051
**AC (** ***n*** ** = 2)**	0/2 (0.0%)	1/2 (50.0%)	1/2 (50.0%)	1/2 (50.0%)	37.5%	1.000
**BC (** ***n*** ** = 65)**	51/65 (78.5%)	50/65 (76.9%)	54/65 (83.1%)	57/65 (87.7%)	81.5%	0.267
**Three columns (ABC) (** ***n*** ** = 127)**	110/127 (86.6%)	117/127 (92.1%)	121/127 (95.3%)	120/127 (94.5%)	92.1%	0.111
**Displacement classification**						
**Displaced injury (** ***n*** ** = 84)**	77/84 (91.7%)	81/84 (96.4%)	81/84 (96.4%)	82/84 (97.6%)	95.5%	0.496
**Non-displaced injury (** ***n*** ** = 223)**	150/223 (67.3%)	167/223 (74.9%)	182/223 (81.6%)	185/223 (83.0%)	76.7%	**0.005**^**#**^

A, B, and C represent medial, intermediate, and lateral column, respectively

^*^The mean value of Observer A’s two observations was compared to that of Observer B

^#^*P* < 0.05

**Fig. 1:**
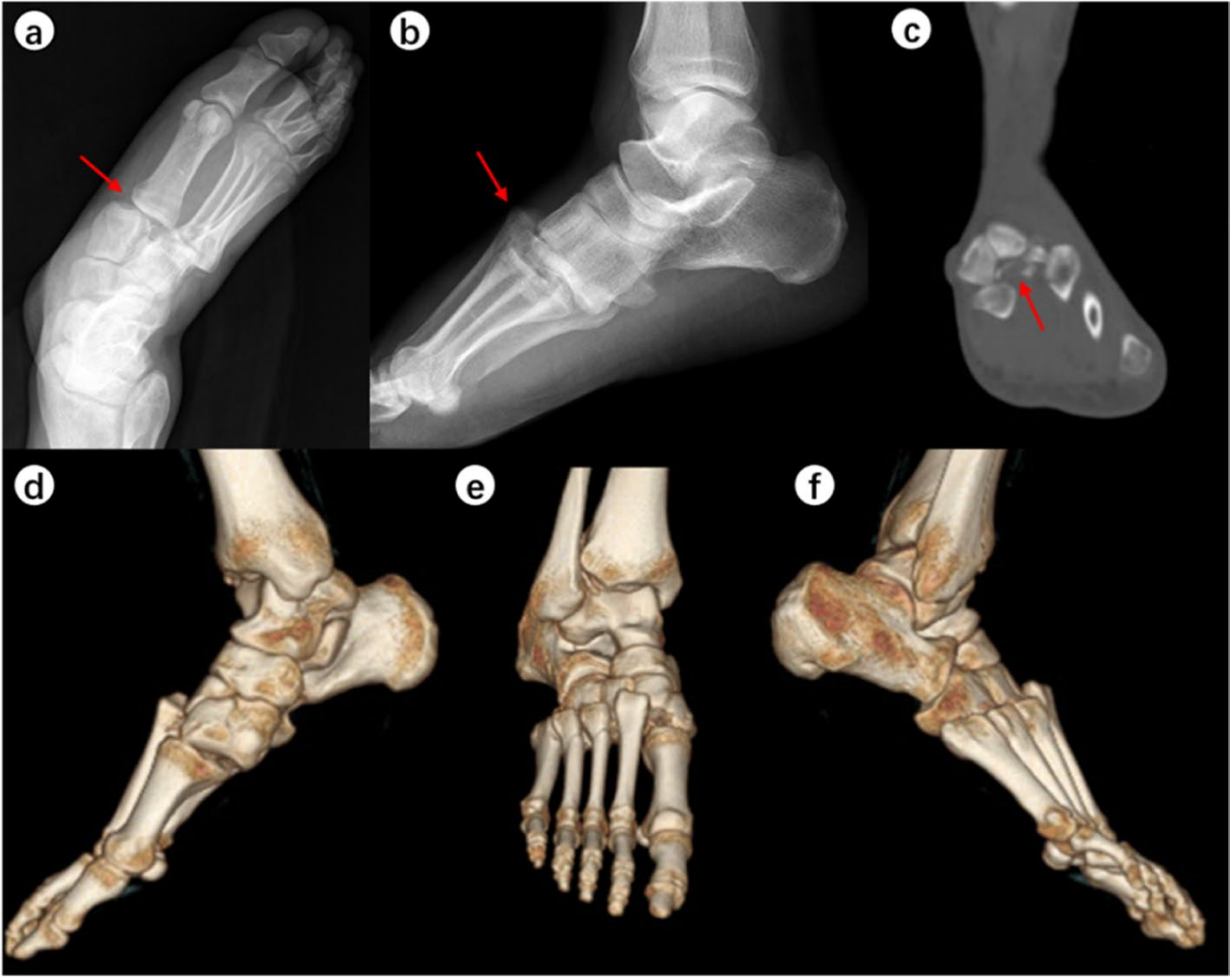
A 42-year-old man had a traffic collision and hurt his right foot. Chiodo-Myerson’s classification: three-column injury; Displacement classification: displaced injury. Both two observers made the correct diagnosis for two times, and didn’t change the initial treatment option (surgery) after evaluating CT image. **a**-**b** The conventional radiographs showed obvious tarsometatarsal joint dislocation (red arrows). It was easily diagnosed. **c**-**f** CT unraveled more details: intra-articular fractures of the base of the second and third metatarsal bone as well as extensive dorsal-lateral dislocation of the tarsometatarsal joint. The red arrow indicates the fracture fragments

**Fig. 2:**
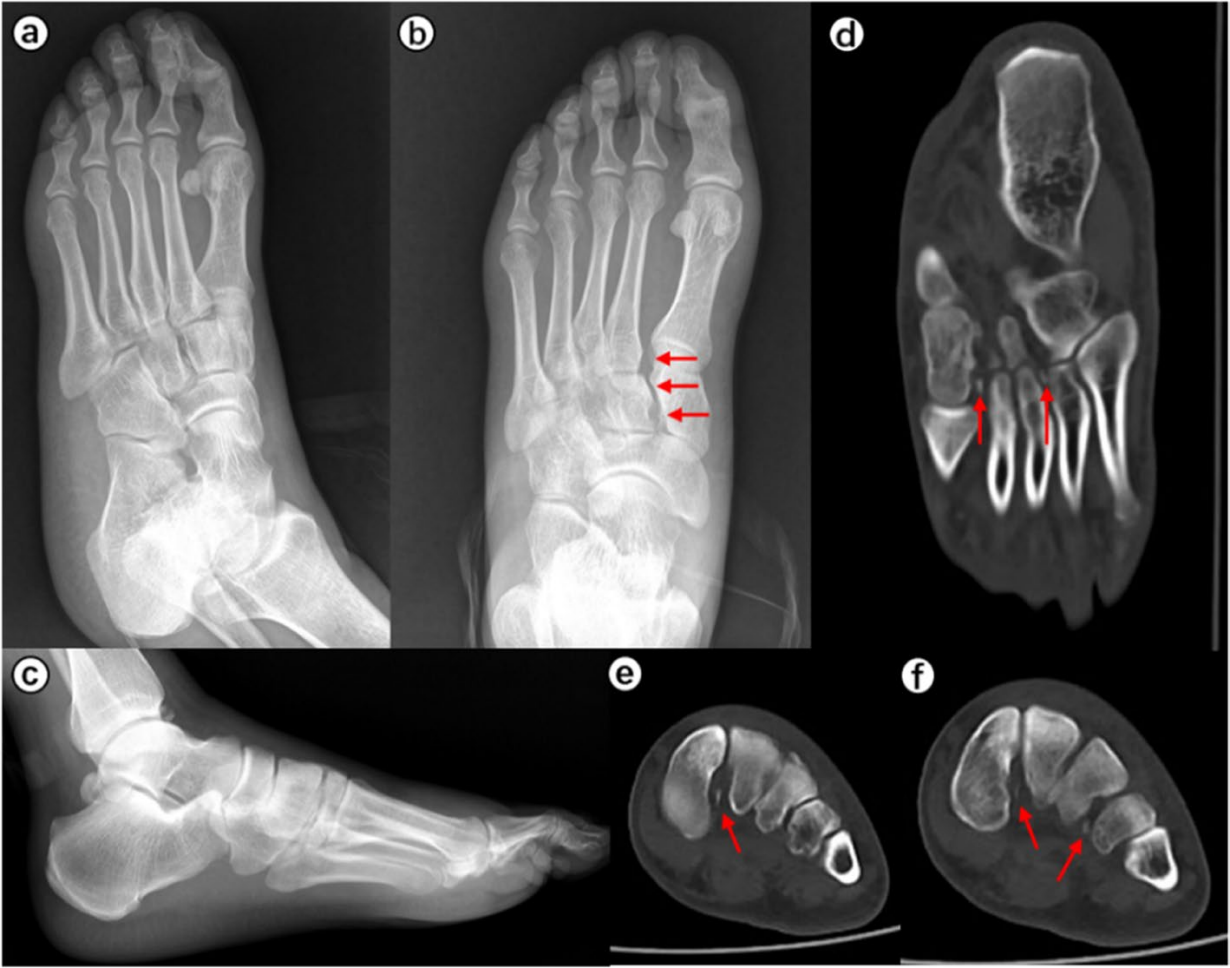
A 37-year-old man fell and hurt his left foot. Chiodo-Myerson’s classification: medial–lateral-column injury; Displacement classification: displaced injury. Both two observers made the correct diagnosis for two times, and didn’t change the initial treatment option (surgery) after evaluating CT image. **a**-**c** The conventional radiographs showed no obvious fractures. The separation of the first and second rays strongly suggested Lisfranc injury (red arrows). **d**-**f** Computed tomography imaging showed intra-articular avulsion fractures of the medial cuneiform and fourth metatarsal bones. Red arrows indicate the fracture fragments

**Fig. 3:**
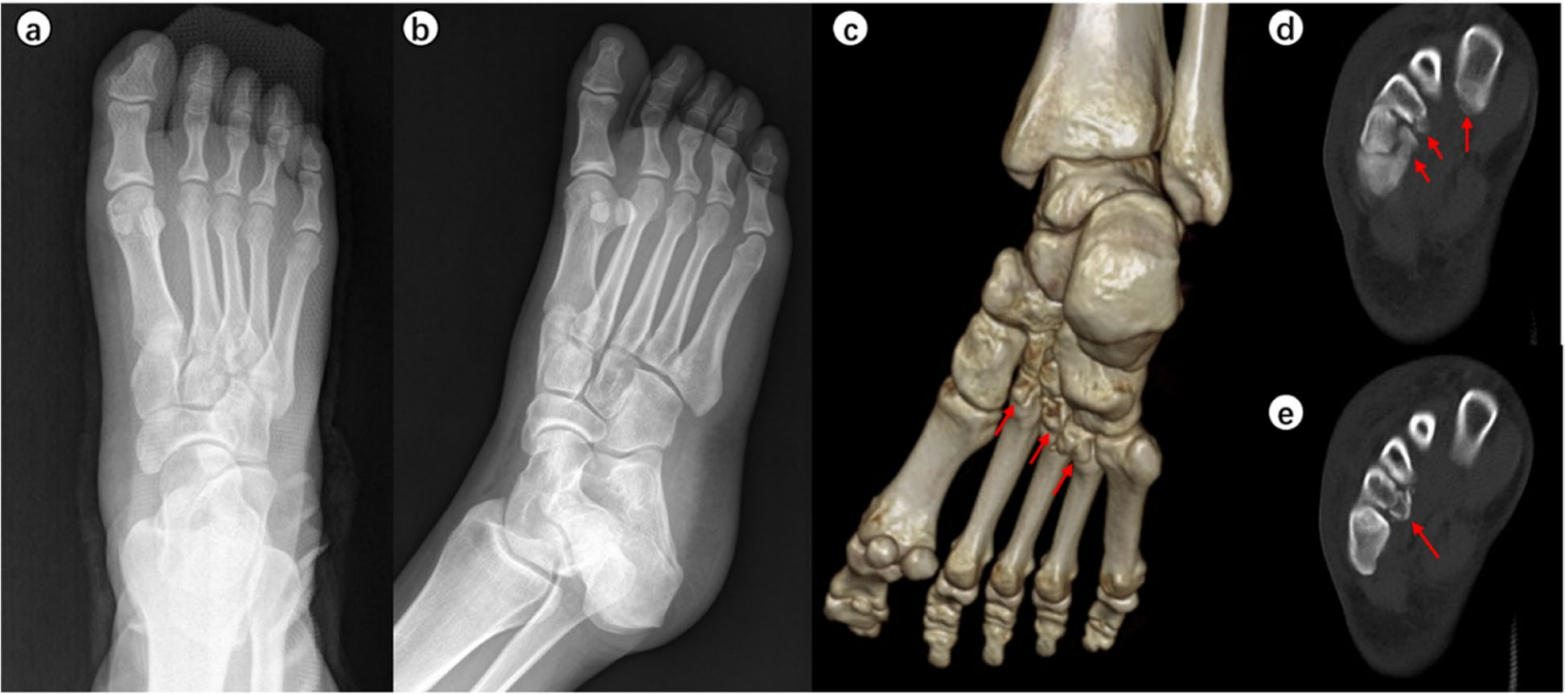
A 30-year-old female hurt her right foot after slipping. Chiodo-Myerson’s classification: three-column injury; Displacement classification: non-displaced injury. Observer A made the wrong diagnosis for two times, and changed the initial treatment option (conservative treatment to surgery) after evaluating CT image. Observer B made the correct diagnosis at the second time, and changed the initial treatment option (conservative treatment to surgery) after evaluating CT image. **a**-**b** The Lisfranc injury was easily missed on plain X-ray. **c**-**e** The plantar intra-articular fractures of the base of the first to fourth metatarsal bones are shown on computed tomography images. The red arrows indicate the fracture fragments

**Fig. 4:**
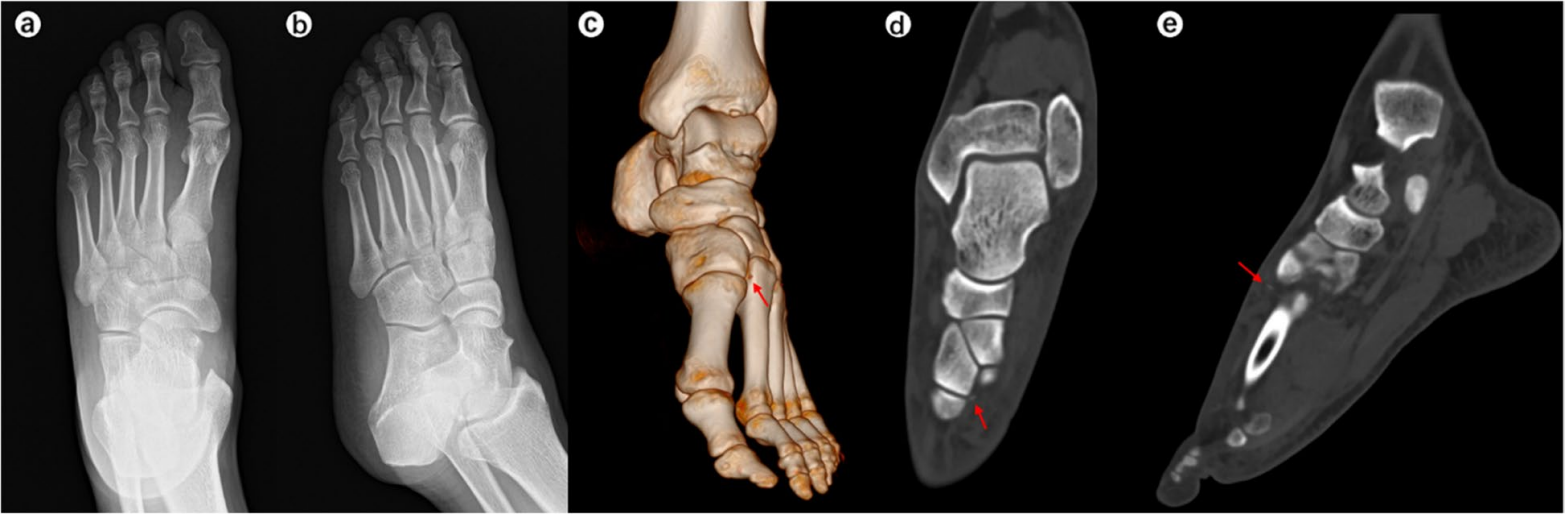
A 46-year-old man slipped and damaged his left foot. Chiodo-Myerson’s classification: medial-column injury; Displacement classification: non-displaced injury. Both two observers made the wrong diagnosis for two times, but didn’t change the initial treatment option (conservative treatment) after evaluating CT image. **a**-**b** The Lisfranc injury was missed on plain X-ray. **c**-**e** Computed tomography showed a dorsal avulsion fracture of the medial cuneiform bone. The red arrows indicate the very small fracture fragments

When the surgeons were asked to re-evaluate their initial treatment strategies after assessing the CT images (Table 5), the mean alteration rate was 21.9% (observer A, 28.3%; observer B, 15.6%). This represented a significant difference in alteration rates between the two observers (*p* < 0.001). Typical case images are shown in the figures (Figs. 1, 2, 3 and 4).

**Table 5:** Whether to change the initial treatment option after evaluating CT images compared with the second conventional radiographs evaluation (*n* = 307)

	**Observer A**	**Observer B**	**Mean value**	***P*** ** value**
Change	87/307 (28.3%)	48/307 (15.6%)	21.9%	** < 0.001**
No change	220/307 (71.7%)	259/307 (84.4%)	78.1%

## Discussion

In this study, we evaluated conventional radiographic images of suspected Lisfranc injuries based on a large sample of consecutive patients. Sensitivity, specificity, and classification accuracy were 81.8%, 90.0%, and 83.8%, respectively. Three-column or displaced injuries had the highest possibility of being recognized. A 21.9% possibility existed of the surgeons changing their initial treatment option after evaluating CT images, after using conventional radiographic images for their first assessments. The diagnostic and clinical decisions made by doctors with different experience levels demonstrated some degree of variability.

Imaging-mediated diagnosis of Lisfranc injury remains challenging because a significant percentage of the injury is non-displaced and even insidious. Imperfection exists in each imaging approach [19]. A recent study showed that bilateral weight-bearing radiographs seemed more valuable than CT scans for diagnosing suspected subtle Lisfranc injuries [20]. Shim et al. argued that the diagnostic validity of bilateral CT is similar to that of bilateral weight-bearing radiographs [21]. Weight-bearing CT, a growing emerging technology, displays a strong potential for detecting subtle changes and revealing latent injuries [22, 23]. Bhimani et al. used three-dimensional volumetric measurements from weight-bearing CT to detect Lisfranc instability with a higher sensitivity (91.6%–92.3%) and specificity (96.5%–97.7%) than those detected using two- and one-dimensional measurements [24]. Despite weight-bearing or manual stress contributing to a higher likelihood of correct diagnosis [25, 26], concomitant pain drastically increased the difficulty in conducting the examinations. Performing such examinations using regional anaesthesia or as follow-up to more conventional methods is likely to be practical and feasible [20, 27].

Magnetic resonance imaging and ultrasonography provide new opportunities in the field, but their utility for detecting Lisfranc injuries requires further investigation. Magnetic resonance imaging has the particular advantages of being highly sensitive and able to reveal occult fractures, and the Lisfranc ligament and other soft tissues [28]; however, it is a time-consuming and costly examination. Raikin et al. found that disruption of the plantar ligament between the first cuneiform and the bases of the second and third metatarsals predicted instability with a sensitivity of 94% and specificity of 75% based on magnetic resonance imaging [29]. Ultrasonography is a cost-effective dynamic diagnostic tool for determining Lisfranc injury by assessing the dorsal Lisfranc ligament [30]. Promoting the use of ultrasonography to diagnose Lisfranc injuries is hampered by a lack of familiarity with the modality among physicians and the technology’s inability to reveal deeper structures.

The majority of patients initially visit primary care doctors, which means that some radiographical methods are not available. Most medical institutions, especially those that provide primary healthcare, prefer conventional radiography. However, this modality’s incidence rates of missed diagnoses and misdiagnosis are unsatisfactory and disappointing. The recognition rate reported in the literature varies from 68.9% to 86.0% [3, 12, 13, 31–34]. Ponkilainen et al. reported the largest sample size [12]. The study included 100 sets of foot radiographs (no Lisfranc injury, non-displaced Lisfranc injury, and displaced Lisfranc injury each representing around 1/3 of the total). The results showed that the overall sensitivity was 76.1% (60.6%–92.4%) and the specificity was 85.3% (52.9%–100%). Furthermore, the diagnostic sensitivity in non-displaced injuries (65.4%) was significantly lower than that in displaced injuries (87.1%).

Our study used the largest data sample thus far reported, reflecting a complicated real-life situation with no manipulation of the proportion of Lisfranc injury subgroups. A total of 223 non-displaced injuries accounted for 72.6% of injuries, which was consistent with the literature (55%–74.7%) [3, 5, 12]. The overall sensitivity and specificity were 81.8% (73.9%–87.0%) and 90.0% (85.0%–94.0%), respectively. We showed a mean recognition rate of non-displaced Lisfranc injuries using conventional radiography of 76.7%, which was remarkably lower than that of the displaced injury group (95.5%). Additionally, column involvement was associated with severity and long-term functional outcomes [35]. We conducted the first subgroup analysis of column involvement: three-column injuries had the highest recognition rate (92.1%), followed by intermediate-lateral-column injuries (81.5%). Interestingly, medial–lateral-column injuries are rare and considered easily ignored. However, the recognition rate of medial–lateral-column injuries may have been affected by the small sample size. Besides, medial-column injuries were relatively difficult to identify (60.7%). Physicians with different levels of experience showed variations in diagnosing non-displaced Lisfranc injuries. The diagnostic rate of non-displaced injuries (76.7%) was significantly lower than that of displaced injuries (95.5%).

Extensive efforts have been made to increase the diagnostic performance of conventional radiography for Lisfranc injuries. Rankine et al. assumed that a craniocaudal angulation of 28.9° might be a better alternative for revealing the Lisfranc joint [13]; bilateral contrast is another potential approach. In contrast to contralateral non-injured side images, Seo et al. compared six abnormal findings in 51 subtle Lisfranc injuries [33]. They recommended medial cuneiform (C1)–second metatarsal (M2) diastasis with a sensitivity of 92% and specificity of 100%.

The reliability of conventional radiography is another concern. Differences among doctors at different experience levels in different departments [12, 32] could affect the reliability of this modality. Sherief et al., reported a mean (range) sensitivity of 86.0% (73.9%–91.3%) and specificity of 92.1% (71.4%–100.0%) using conventional radiographs detecting Lisfranc injuries [32]. However, the intraclass correlation coefficient was not further calculated. Ponkilainen et al. reported that the mean (range) sensitivity was 76.1% (60.6%–92.4%) and the specificity was 85.3% (52.9%–100.0%) [12]. Further results for interobserver and intra-observer reliability confirmed moderate to substantial correlation (mean κ = 0.54 and 0.71, respectively). In our study, the mean (range) sensitivity was 81.8% (73.9%–87.0%) and specificity was 90.0% (85.0%–94.0%). The mean κ for interobserver reliability was 0.69 and intra-observer reliability was 0.73, which resulted in a substantial correlation.

More importantly, our study is the first to explore clinical decision-making based on the use of conventional radiography versus CT. The mean alteration rate was 21.9%, with the senior surgeon demonstrating a lower tendency to alter their decision (15.6%) than the junior surgeon did (28.3%). Based on this observation, new questions can be formulated: How do we reduce lapses in clinical decision-making processes? When and how should we choose further investigation using other imaging modalities? These questions warrant consideration and future research.

### Limitations of this study

This study has several limitations. First, two foot and ankle surgeons, considered necessarily representative, participated in this assessment; findings based on their contributions to this study might not be generalizable or representative of broader patterns. The findings need to be interpreted and extrapolated with caution. Second, a relatively large sample was included in our study to reflect a real situation; however, the sample size of some uncommon types of Lisfranc injuries was small, thus limiting the related subgroup analyses. Third, the complexity of specific treatments meant that we only compared two general management options – surgery and conservative treatment.

## Conclusion

The sensitivity, specificity, and classification accuracy of conventional radiographs for Lisfranc injuries were 81.8%, 90.0%, and 83.8%, respectively. Three-column or displaced injuries had the highest likelihood of being recognized. The possibility of changing the initial treatment option after evaluating CT images compared to conventional radiographs was 21.9%. Furthermore, the diagnosis and clinical decisions made by doctors with different seniority levels demonstrated some degree of variability. Protected weight-bearing and a further CT scan should be considered for patients with positive signs in physical examination, but negative findings in conventional radiography.

## Data Availability

The datasets generated and analyzed during the current study are available from the corresponding author on reasonable request.

## Abbreviations

CT: Computed tomography
TPR: True positive rate
TNR: True negative rate
FPR: False positive rate
FNR: False negative rate
PPV: Positive predictive value
NPV: Negative predictive value
ACC: Classification accuracy
BER: Balanced error rate
